# Request–response characteristics and public satisfaction with using a health hotline

**DOI:** 10.3389/fdgth.2026.1702945

**Published:** 2026-02-13

**Authors:** ShuHan Sun, JingFu Lai, Juan Wang, Xue Bai, ZhiWei Wang, RuQing Liu, RuWei Hu

**Affiliations:** 1 Department of Health Management, School of Public Health, Sun Yat-Sen University, Guangzhou, China; 2 Guangdong Provincial Engineering Technology Research Center of Environmental Pollution and Health Risk Assessment, Department of Occupational and Environmental Health, School of Public Health, Sun Yat-Sen University, Guangzhou, China; 3 12320 Health Hotline, Guangzhou Center for Disease Control and Prevention, Guangzhou, China; 4 School of Law, Sun Yat-Sen University, Guangzhou, China

**Keywords:** health hotline, medical quality, patient satisfaction, telehealth, telemedicine, telephone

## Abstract

**Background:**

Health hotlines serve as platforms for patient–provider communication globally. However, few studies have explored public satisfaction with health hotline services. This study investigated the associations between the characteristics of health hotline platforms and public satisfaction with their services, aiming to provide insights for optimizing risk communication and improving residents’ access to health information via health hotlines.

**Methods:**

A retrospective observational study was conducted using calls and messages (CMs) received on a health hotline platform in Guangzhou, China, in 2023 and 2024. Generalized linear models were used to assess the associations between various request–response characteristics and public satisfaction with the hotline service.

**Results:**

A total of 9,280 satisfaction-related CMs were included in the analysis. All the request–response characteristics of the hotline were associated with public satisfaction. For example, responses at the department level were more satisfying than those at the hospital level, with an odds ratio (OR) of 1.26 (95% CI 1.05–1.51). Regarding CM content, CMs related to patient safety had a lower satisfaction than non-clinical CMs, with an OR of 0.60 (95% CI 0.45–0.78). Additionally, as the response time increased, residents’ satisfaction tended to decrease (*P* for trend <0.05).

**Conclusions:**

The study indicated that request–response characteristics, such as response hierarchy and response time, significantly influence public satisfaction with health hotlines. Our findings provide novel evidence for optimizing the service quality of health hotline platforms to improve public satisfaction, which is valuable for enhancing patient–provider communication and improving the efficiency and quality of healthcare delivery.

## 1 Introduction

Effective communication has been identified as an important factor in healthcare delivery and significantly influences public satisfaction (1). Many countries and agencies have implemented health hotline platforms as a foundational element of their modern digital health systems (2–7). Telephone-based services such as health hotlines constitute a primary form of “client-to-provider telemedicine,” facilitating remote triage and serving as an inclusive entry point to the health system that bridges the digital divide (8). Some health hotlines have been proven to be effective in enhancing communication effects and public satisfaction (9–11). For example, a study in Scotland investigated the public's use of and attitude towards NHS 24, the NHS hotline, and revealed that over 80.0% of callers were satisfied with the NHS 24 service and that 93.9% would use it again; thus, NHS 24 has been viewed as an out-of-hours alternative to general practitioners (9). A systematic review from Australia reported that 988 cancer helplines deliver psychosocial benefits to patients affected by cancer, and the majority of cancer helpline callers were generally satisfied with the information they received, the way their call was managed, and the consultants’ knowledge and approach (4).

Thus, optimizing hotline operations is not merely a service improvement but a strategic enhancement of digital health infrastructure. Exploring the operational mechanism and core function of health hotline platforms is highly important for offering actionable insights to increase public satisfaction and improve patient–provider communication. A study from Oman assessed satisfaction with telephone-based psychiatry consultations and found that the sex, employment status, and income of users significantly influenced satisfaction levels (12). However, beyond demographic factors, understanding how platform-specific characteristics affect user experience is crucial. Evidence regarding the specific request–response characteristics of health hotline platforms that influence satisfaction remains scarce. Crucially, studies have found that actions taken by health hotline platforms, such as conducting training for hotline staff (13,14), enhancing communication competence, and promoting systems-based practice (14), are effective in improving communication via health hotlines.

Yet, research on the request–response characteristics of health hotline platforms that affect public satisfaction is still lacking, especially in developing countries. In the present study, we explored the associations between the characteristics of health hotline services and public satisfaction based on residents’ calls and messages (CMs).

## 2 Methods

### 2.1 Study setting and patient and public involvement

The 12320 health hotline is a platform that was established by the National Health Commission of the People's Republic of China. Originating from the Severe Acute Respiratory Syndrome (SARS) Prevention and Consultation Hotline during the 2003 SARS outbreak, 12320 was officially launched in 2005 and implemented nationwide by 2012, functioning as a governmental conduit for health-related communication between the public and healthcare providers.

This study was conducted on a dataset from the 12320 Health Hotline Department of the Guangzhou Center for Disease Control and Prevention. Guangzhou is a core city in the Guangdong–Hong Kong–Macao Greater Bay Area in China.

The 12320 Health Hotline in Guangzhou (12320-GZ) serves as a governmental health hotline platform for communication between the public and healthcare providers and guides residents’ CMs to relevant institutions, enhances hospital supervision, and addresses public concerns effectively.

12320-GZ staff respond to residents’ health requests in various ways, such as providing information and advice on appropriate self-care, arranging callbacks or visits by relevant clinical professionals, or referring them to other services when necessary.

### 2.2 Data source

CM data were collected from the 12320-GZ platform in 2023 and 2024. Then, we excluded CMs with missing satisfaction data. The response rate for the public satisfaction survey was 34.4%. In total, we gathered 9,280 CMs.

### 2.3 Identification of variables

We collected data on public satisfaction and request–response characteristics from the 12320-GZ system.

#### 2.3.1 Outcome variable

Public satisfaction was initially rated on a 5-point scale (ranging from “very dissatisfied” to “very satisfied”). To facilitate model parsimony and provide a clear interpretation of the primary drivers of satisfaction, we dichotomized this variable. “Very satisfied” and “satisfied” were rated as 1 (satisfied), while the remaining grades were rated as 0 (dissatisfied). To ensure that this loss of granularity did not distort the findings, we performed a sensitivity analysis using multinomial logistic regression on the original 5-point scale, which confirmed the consistency of the results. Satisfaction ratings were self-reported by the residents and uploaded to the 12320-GZ system (see Supplementary Material).

#### 2.3.2 Explanatory variables

The request–response characteristics included the following variables. Detailed definitions are provided in Table 1.

(1) Response hierarchy: The organizational level at which the CM was processed and resolved (classified as either the general hospital level or the specific department level).
(2) Response mode: The communication channel utilized by the platform to deliver the final feedback to the resident (e.g., telephone, message, or alternative means).
(3) Resident's request: The primary intent or category of the resident's interaction (classified as a suggestion, consultation, or complaint).
(4) CM content: The specific subject matter or nature of the issue raised in the CM (e.g., clinical quality or patient safety).
(5) Facticity verification: The extent to which the details described by the resident were verified and acknowledged as accurate by the healthcare provider following an internal investigation (classified as accepted, partially accepted, or not accepted).
(6) Response time: The total duration from when the platform received the CM to the resident receiving the response. This was categorized into the following four quartiles: Q1 (0–3 days), Q2 (3–9 days), Q3 (9–13 days), and Q4 (13–48 days).

**Table 1:** Variables and variable levels of the request–response characteristics provided by 12320-GZ.

Variable	Variable levels and description
Response hierarchy	Hospital level: CMs processed at the hospital level
	Department level: CMs processed at the specific hospital department
Response time	The following four quantiles were divided according to the response time (the time from when 12320-GZ received the CMs to when the patient got the response from 12320-GZ): quantile 1 (Q1, ≤25th): 0–3days;
	quantile 2 (Q2, 25th–50th): 3–9 days;
	quantile 3 (Q3, 50th–75th): 9–13 days;
	quantile 4 (Q4, ≥75th): 13–48 days
Resident's request	Suggestion: CM providing constructive opinions or advice to improve healthcare services
	Consultation: CM requesting health-related information or guidance
	Complaint: CM expressing dissatisfaction or criticism about perceived issues, mistakes, or misconduct in healthcare settings
Response mode	Telephone response: responded via phone call.
	Message response: responded via short message service
	Alternative response: other types of response to the patient, such as a written response
CM content	Non-clinical item: CM related to administrative matters that did not involve medical or nursing matters, such as appointment scheduling and health certificate requests
	Systemic issue: CM related to organizational and systemic issues affecting the overall functioning of healthcare services, instead of specific incidents, such as a staff shortage, long wait time, inefficiencies in workflows, and inadequate facilities
	Clinical quality: CM related to medical quality outcome from the resident’s perspective during healthcare experiences that were not a severe safety risk, such as dissatisfaction with treatment effectiveness and unsuccessful communication with healthcare staff
	Patient safety: CM related to serious incidents or concerns threatening patient safety, such as medication errors, surgical complications, adverse drug reactions, and healthcare-associated infections
Facticity verification	Not accepted: not accepted due to the resident’s entirely inaccurate or unreasonable description
	Partially accepted: partially accepted due to a partially inaccurate or unreasonable description
	Accepted: accepted due to accurate verification by medical providers

### 2.4 Statistical analysis

Categorical variables are expressed as frequencies and percentages *n* (%). Pearson's chi-square test was used to compare baseline characteristics between the dissatisfied and satisfied groups.

**Figure 1:**
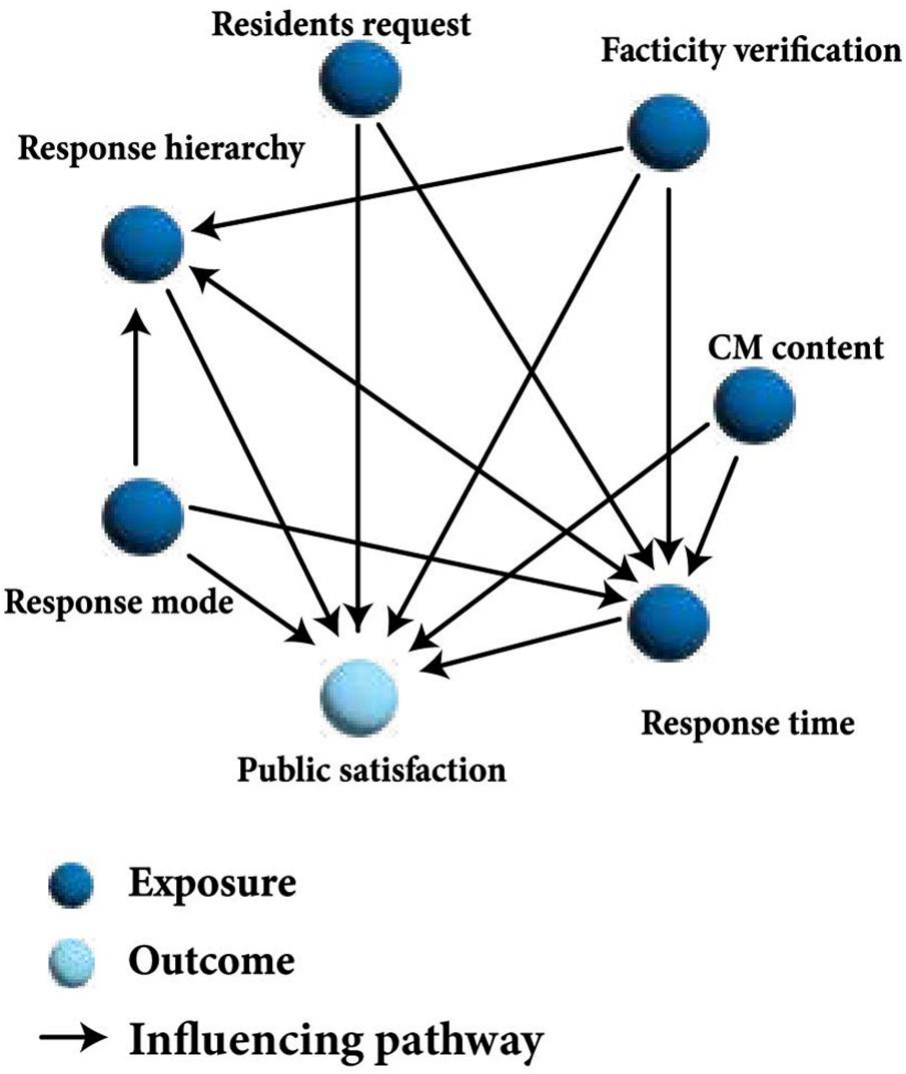
The DAG of the relationship between the request–response characteristics of the health hotline platform and public satisfaction.

A directed acyclic graph (DAG) was constructed to guide the selection of potential confounders and illustrate the hypothesized causal pathways between the request–response characteristics and public satisfaction (Figure 1). The structure of the DAG was informed by prior subject matter knowledge, a literature review, and consultations with health hotline staff. To validate the robustness of the DAG-informed adjustment strategy, we performed a sensitivity analysis using a series of six nested models.

Associations were analyzed using generalized linear models. Based on the DAG analysis, the variables used in the model included the response hierarchy, response time, resident's request, response mode, CM content, and facticity verification (Figure 1). In addition, the *P*-value for the trend was calculated for response time. Results are presented as odds ratios (ORs) with 95% confidence intervals (CIs).

*P* < 0.05 was considered statistically significant. All analyses were performed using R software (version 4.1.2).

## 3 Results

### 3.1 Characteristics of the 12320-GZ health hotline

The characteristics of the 9,280 analyzed CMs are summarized in Table 2. Overall, 65.84% of the residents were satisfied with the service. The majority of the CMs were processed at the hospital level (83.2%), were complaints (64.1%), and were related to clinical quality (56.0%). Significant differences were observed across all request–response variables between the satisfied and dissatisfied groups (*P* < 0.001 for all comparisons), indicating that satisfaction was highly sensitive to platform operational characteristics (Table 2).

**Table 2:** Analysis of the CMs.^a^

Variable	Total (*N* = 9,280)	Dissatisfied (*N* = 3,170, 34.16%)	Satisfied (*N* = 6,110, 65.84%)	*P-*value
Response hierarchy				**<0.001**
Hospital level	7,721 (83.2%)	2,802 (88.4%)	4,919 (80.5%)	
Department level	1,559 (16.8%)	368 (11.6%)	1,191 (19.5%)	
Response time				**<0.001**
Q1	2,782 (30.0%)	491 (15.5%)	2,291 (37.5%)	
Q2	2,086 (22.5%)	553 (17.4%)	1,533 (25.1%)	
Q3	2,360 (25.4%)	1,041 (32.8%)	1,319 (21.6%)	
Q4	2,052 (22.1%)	1,085 (34.2%)	967 (15.8%)	
Resident's request				**<0.001**
Suggestion	400 (4.3%)	113 (3.6%)	287 (4.7%)	
Consultation	2,933 (31.6%)	286 (9.0%)	2,647 (43.3%)	
Complaint	5,947 (64.1%)	2,771 (87.4%)	3,176 (52.0%)	
Response mode				**<0.001**
Telephone response	4,664 (50.3%)	875 (27.6%)	3,789 (62.0%)	
Message response	4,142 (44.6%)	2,097 (66.2%)	2,045 (33.5%)	
Alternative response	474 (5.1%)	198 (6.2%)	276 (4.5%)	
CM content				**<0.001**
Non-clinical item	1,113 (12.0%)	235 (7.4%)	878 (15.3%)	
Systemic issue	1,114 (12.0%)	247 (7.8%)	867 (13.8%)	
Clinical quality	5,197 (56.0%)	1,791 (56.5%)	3,406 (55.7%)	
Patient safety	1,856 (20.0%)	897 (28.3%)	959 (15.2%)	
Facticity verification				**<0.001**
Not accepted	2,274 (24.5%)	1,381 (43.6%)	893 (14.6%)	
Partially accepted	3,100 (33.4%)	1,208 (38.1%)	1,892 (31.0%)	
Accepted	3,906 (42.1%)	581 (18.3%)	3,325 (54.4%)	

Bold text indicates that the associations were statistically significant at *P* < 0.05.

a Values are expressed as the number of cases (percentages). Statistical comparisons between the satisfied and dissatisfied groups were performed using Pearson's chi-square test. All the statistical tests were two-sided, with the significance level set at *P* < 0.05.

### 3.2 Request–response characteristics associated with public satisfaction with health hotlines

**Figure 2:**
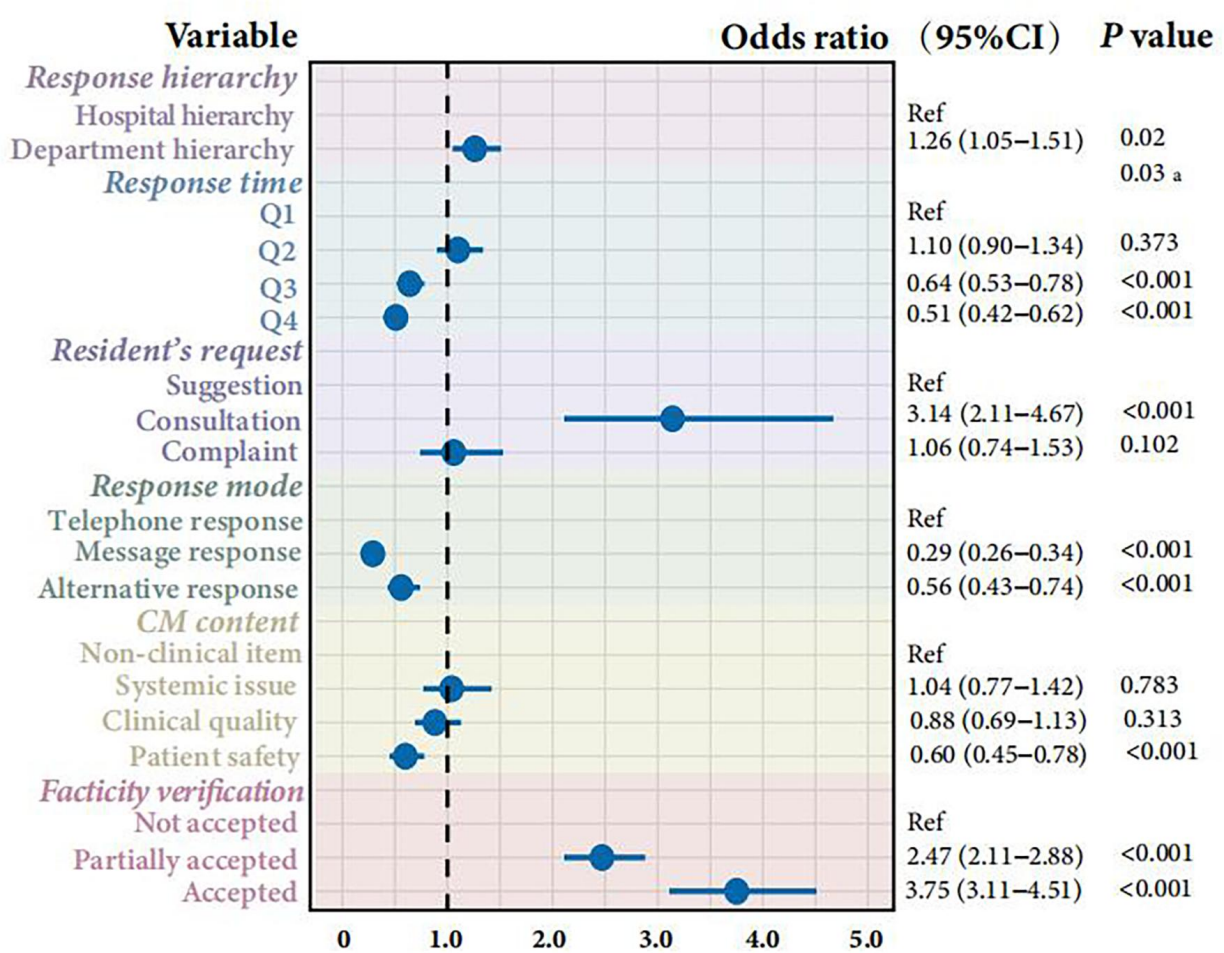
Forest plots of the associations between the request–response characteristics and public satisfaction. *P*-value for trend that was tested by including the median of each quartile of response time as a continuous variable in the models.

The multivariable logistic regression analysis revealed significant associations between platform characteristics and public satisfaction (Figure 2). CMs processed at the department level were significantly associated with higher satisfaction compared to those processed at the general hospital level (OR = 1.26, 95% CI 1.05–1.51), suggesting that specialized handling improves user experience. CMs involving patient safety were associated with a 40% lower likelihood of satisfaction compared to non-clinical items (OR = 0.60, 95% CI 0.45–0.78), highlighting patient safety as a critical area of contention. We observed a significant inverse trend for response time, with public satisfaction notably decreasing as the response time increased (*P* for trend <0.05). Specifically, compared to the quickest response time (Q1), response times in the third (Q3) and fourth (Q4) quartiles were associated with significantly lower odds of satisfaction. Verification status was the strongest predictor. CMs that were “accepted” (fully verified) had significantly higher odds of satisfaction (OR = 3.75, 95% CI 3.11–4.51) compared to those that were not accepted.

## 4 Discussion

### 4.1 Summary

This study revealed that the level of satisfaction with this health hotline platform was higher when CMs were handled at the departmental level, were responded to via telephone, were addressed promptly, and were properly verified. However, CMs involving patient safety were associated with lower satisfaction. To date, our study is the first globally to report on the associations between the request–response characteristics of health hotline platforms and public satisfaction, providing empirical evidence for optimizing digital health governance.

### 4.2 Satisfaction and response hierarchy

This study revealed that directing CMs to specific departments rather than general management offices increases public satisfaction. This finding aligns with international triage models, such as the UK's NHS 111 service, which utilizes the “NHS Pathways” system to escalate complex clinical cases from call handlers to clinical advisors or specialists (15). This tiered response mechanism ensures that patients receive care appropriate to the urgency and complexity of their condition.

Assigning CMs to specific departments in hospitals promotes a more targeted and precise process. This reflects the core competency of “systems-based practice,” which requires professionals to understand the larger organizational context and effectively mobilize resources to provide optimal coordination of care (16). Therefore, training for hotline staff should extend beyond clinical knowledge to include competencies in health system navigation and resource allocation. Policymakers should develop strategies to regulate institutional standards and ensure that residents’ inquiries reach the most appropriate provider efficiently, thereby building trust in the digital health infrastructure.

### 4.3 Satisfaction and response time

Our study revealed that satisfaction increased when response times were quicker than 10 days but dropped significantly after 13 days. This “satisfaction window” highlights the urgency of efficient triage.

However, not every CM can be resolved quickly, especially those involving complicated investigations. Research on healthcare complaints indicates that patients often perceive unexplained delays as institutional apathy rather than procedural necessity (17). The “Principles of Good Complaint Handling” by the UK Parliamentary and Health Service Ombudsman suggests that keeping the caller informed of the investigation status is a proven strategy to manage expectations and maintain trust, even when the resolution is delayed (18).

### 4.4 Satisfaction and CM content

Our study found that CMs related to patient safety had the lowest public satisfaction. This likely stems from the high emotional stakes and complexity inherent in safety incidents. Complaints involving safety often reflect breakdowns in care or medical errors, leading to patient distress that is difficult to alleviate through standard hotline responses.

This finding has profound implications for medical education and staff training. According to the WHO’s Multi-professional Patient Safety Curriculum Guide, effective communication is crucial when managing safety incidents (17). To improve satisfaction in this domain, we propose the following three educational priorities for hotline staff.

First, staff require training in active listening and de-escalation techniques to handle distressed callers. Research has consistently shown that empathy is strongly correlated with patient satisfaction and can mitigate the negative impact of adverse events (19). Second, hotline staff serve as the “sensor” for system-wide risks and, thus, training should empower them to identify “red flag” safety issues immediately and trigger appropriate escalation protocols, rather than offering generic responses. Third, for management, low satisfaction in safety cases suggests a need for systemic learning. Platforms could utilize root cause analyses of these complaints to improve quality, transforming individual grievances into organizational learning (20).

## 5 Strengths and limitations

This study offers a unique research perspective by analyzing the associations between request–response characteristics and public satisfaction with a health hotline in a large sample from Guangzhou, China. These findings provide a paradigm for improving communication via health hotlines.

However, several limitations should be acknowledged. First, as a core city in the Guangdong–Hong Kong–Macao Greater Bay Area, Guangzhou's large population made the data valuable for gaining insights to enhance the quality of health hotlines and public satisfaction. However, the findings may not represent the experiences of all residents across the country, especially those living in rural or less developed regions who may lack access to official hotline platforms or have different health concerns.

Second, the response rate for the satisfaction survey was 34.4%. Although this response rate may introduce potential non-response bias, the substantial sample size (*n* = 9,280) provides adequate statistical power for the analysis.

Third, owing to the protection of residents’ personal privacy, we were not able to collect data on the individual demographic characteristics of the respondents, which may influence individual satisfaction levels. Given that our study focuses on the impact of the platform's request–response factors on public satisfaction, the absence of individual-level data does not significantly undermine the validity of the study.

## 6 Conclusion

Our findings establish the critical role of request–response characteristics in determining public satisfaction with health hotline platforms, offering novel evidence for optimizing digital health governance. Practically, to enhance service quality, healthcare administrators should prioritize establishing specific departmental workflows for complex clinical cases rather than general handling. Furthermore, hotline staff require specialized training in patient safety competencies and active verification protocols to manage the high expectations associated with clinical inquiries. Further research is needed to develop standardized benchmarks for health hotline operational mechanisms, ensuring that communication is not only timely but also targeted, articulated, and effective.

## Data Availability

The data analyzed in this study are subject to the following licenses/restrictions: the data that support the findings of this study are available from the 12320 Health Hotline Department of the Guangzhou Center for Disease Control and Prevention, but restrictions apply to the availability of these data, which were used under license for the current study and are not publicly available. Data are, however, available from the authors upon reasonable request and with permission from the 12320 Health Hotline Department of the Guangzhou Center for Disease Control and Prevention. Requests to access these datasets should be directed to JW at 342133376@qq.com.
