# Supplementary material for: Request–response characteristics and public satisfaction with using a health hotline
Source: Front Digit Health. 2026 Feb 13;8:1702945. doi: 10.3389/fdgth.2026.1702945 (PMC12946935; doi:10.3389/fdgth.2026.1702945)
Supplement: Supplementary file 1 [file Table1.docx]

Table S1 Multiple logistic regression results

| Variables | Very dissatisfied | Dissatisfied | Generally | Satisfied | Very satisfied |
| --- | --- | --- | --- | --- | --- |
| **Response hierarchy** |  |  |  |  |  |
| Hospital hierarchy | Ref | Ref | Ref | Ref | Ref |
| Department hierarchy | Ref | **1.73 (1.25, 2.39)** | **1.57 (1.21, 2.01)** | **1.32 (1.02, 1.70)** | **1.39 (1.11, 1.74)** |
| **Response time** |  |  |  |  |  |
| Q1 | Ref | Ref | Ref | Ref | Ref |
| Q2 | Ref | 1.13 (0.62, 1.38) | 1.23 (0.79, 2.19) | 133. (0.94, 2.13) | 1.17 (0.92, 1.50) |
| Q3 | Ref | 1.04 (0.73, 1.48) | 0.85 (0.61, 1.13) | 0.94 (0.71, 1.24) | **0.64 (0.50, 0.80)** |
| Q4 | Ref | 0.86 (0.59, 1.22) | **0.74 (0.55, 0.99)** | **0.66 (0.49, 0.89)** | **0.41 (0.32, 0.52)** |
| **Resident’s request** |  |  |  |  |  |
| Suggestion | Ref | Ref | Ref | Ref | Ref |
| Consultation | Ref | 0.64 (0.21, 1.28) | 1.49 (0.80, 2.76) | **2.15 (1.27, 3.84)** | **3.16 (1.89, 5.27)** |
| Complaint | Ref | 0.53 (0.37, 1.12) | 1.00 (0.56, 1.77) | 0.77 (0.46, 1.31) | 0.67 (0.41, 1.08) |
| **Response mode** |  |  |  |  |  |
| Telephone response | Ref | Ref | Ref | Ref | Ref |
| Message response | Ref | 1.07 (0.83, 1.38) | **0.31 (0.26, 0.38)** | **0.31 (0.28, 0.38)** | **0.29 (0.24, 0.34)** |
| Alternative response | Ref | 1.15 (0.71, 1.84) | **0.67 (0.45, 0.97)** | **0.57 (0.38, 0.85)** | **0.59 (0.41, 0.82)** |
| **CM content** |  |  |  |  |  |
| Nonclinical item | Ref | Ref | Ref | Ref | Ref |
| Systemic issue | Ref | 0.65 (0.42, 1.28) | 0.93 (0.65, 1.32) | 0.74 (0.53, 1.03) | 0.70 (0.52, 1.53) |
| Clinical quality | Ref | 0.68 (0.46, 1.15) | 0.96 (0.61, 1.48) | 0.92 (0.61, 1.37) | 0.79 (0.55, 1.12) |
| Patient safety | Ref | **0.62 (0.39, 0.98)** | **0.67 (0.43, 0.92)** | **0.51 (0.35, 0.74)** | **0.42 (0.36, 0.58)** |
| **Facticity verification** |  |  |  |  |  |
| Not accepted | Ref | Ref | Ref | Ref | Ref |
| Partially accepted | Ref | 0.98 (0.74, 1.27) | **2.6 (2.05, 3.27)** | **2.84 (2.22, 3.61)** | **2.12 (1.74, 2.58)** |
| Accepted | Ref | 1.08 (0.76, 1.50) | **3.7 (2.83, 4.83)** | **4.37 (3.33, 5.71)** | **3.66 (2.93, 4.57)** |

Bold text indicates that the associations were statistically significant at *P* < 0.05.
